# How a face may affect object-based attention: evidence from adults and 8-month-old infants

**DOI:** 10.3389/fnint.2014.00027

**Published:** 2014-03-26

**Authors:** Eloisa Valenza, Laura Franchin, Hermann Bulf

**Affiliations:** ^1^Dipartimento di Psicologia dello Sviluppo e della Socializzazione, Università degli Studi di PadovaPadova, Italy; ^2^Interdepartmental Center for Cognitive Science, Università degli Studi di PadovaPadova, Italy; ^3^Dipartimento di Psicologia, Università degli Studi di Milano-BicoccaMilano, Italy

**Keywords:** object-based attention, visual attention, faces, eye-tracker, infancy

## Abstract

Object-based attention operates on perceptual objects, opening the possibility that the costs and benefits humans have to pay to move attention between-objects might be affected by the nature of the stimuli. The current study reported two experiments with adults and 8-month-old infants investigating whether object-based-attention is affected by the type of stimulus (faces vs. non-faces stimuli). Using the well-known cueing task developed by Egly et al. (1994) to study the object-based component of attention, in Experiment 1 adult participants were presented with two upright, inverted or scrambled faces and an eye-tracker measured their saccadic latencies to find a target that could appear on the same object that was just cued or on the other object that was uncued. Data showed that an object-based effect (a smaller cost to shift attention within- compared to between-objects) occurred only with scrambled face, but not with upright or inverted faces. In Experiment 2 the same task was performed with 8-month-old infants, using upright and inverted faces. Data revealed that an object-based effect emerges only for inverted faces but not for upright faces. Overall, these findings suggest that object-based attention is modulated by the type of stimulus and by the experience acquired by the viewer with different objects.

## INTRODUCTION

Selective attention allows us to pick out and respond to relevant information while ignoring the myriad distracting stimuli in cluttered visual scenes. Attention selects on the basis of spatial location (space-based attention), but the unit of attentional selection may also be based on objects (object-based attention), in addition to space. Space-based attention facilitates responses to the stimuli within the selected area of the visual field (Posner, 1980; Posner et al., 1980; Cave and Bichot, 1999), whereas object-based attention facilitates selection of whole objects (Duncan, 1984; Driver and Baylis, 1998; Driver et al., 2001; Scholl, 2001).

The best-known demonstration of object-based attention is that of Duncan (1984), but after this first study there has been an explosion of adults’ research on object-based selection (for a reviews see, Kanwisher and Driver, 1992; Driver and Baylis, 1998; Scholl, 2001). The research conducted by Egly et al. (1994) is of particular significance for the current study because it introduced a paradigm that has been recently adapted to investigate the object-based component of attention even in infancy (Bulf and Valenza, 2013). Participants were presented with two rectangles. One end of a rectangle was cued immediately before the presentation of a target. Valid targets appeared at the cued location. Invalid targets could appear either in the other end of the cued rectangle (within object), or in the other rectangle, but at the same distance from the cue (between-object). Target detection was faster for valid targets than for invalid ones (space-based effect). Moreover, detection was faster for invalid within-object trials, than for invalid between-object trials (object-based effect). The object-based effect provides evidence that objects can affect the distribution of attention and that attending to one aspect of an object facilitates the processing of other aspects of the same object.

Using variants of Egly et al.’s (1994) paradigm, it was demonstrated that several factors may affect the presence or the absence of the object effect (Macquistan, 1997; Goldsmith and Yeari, 2003; Li and Logan, 2008; Chen, 2012). For instance, recently Chen (2012) has demonstrated that the *goodness* of an object (i.e., Gestalt principles, such as continuation, collinearity, or common fate) influences the degree to which object-based attention is utilized suggesting that a critical factor in eliciting the deployment of object-based attention is the establishment of a viable representation.

Starting from these findings the first aim of the current paper was to investigate whether adults’ object-based attention is affected by the stimulus type: Do faces modulate the object-based component of visual attention differently from non-face objects? Several adult studies have already revealed that in adulthood a face can orient visual attention differently by a non-face stimulus (e.g., Bindemann et al., 2005, 2007; Hershler and Hochstein, 2005; Theeuwes and Van der Stigchel, 2006; Langton et al., 2008; Taylor and Therrien, 2008). For example, when a visual search task was used to determine the nature of face perception, it has been shown that a face pops out when it was presented among non-face distractor objects (Hershler and Hochstein, 2005), but not when it was presented among inverted faces (Kuehn and Jolicoeur, 1994; Brown et al., 1997). However, when distractors looked less like faces, search for a face became easier (Kuehn and Jolicoeur, 1994).

Here we investigated the effect of faces on visual attention using a task that is considered a direct measure of the object-based component of visual attention, that is the Egly et al.’s (1994) cueing task. Adults were presented with two upright, inverted or scrambled faces and an eye-tracker system measured the saccadic latency toward a target that could appeared in a previously cued location (valid condition; VAL), in an uncued location of the cued object (invalid same-object condition; ISO), or in a location of the uncued object placed at the same distance from the cue as the target in the ISO (invalid different-object condition; IDO; Experiment 1). Participants were told simply to look at the display, without any additional instruction. The measure of saccade latency in a free looking condition (i.e., without any verbal instructions) is a suitable tool for investigating the object-based component of attention and has the advantage to permit the use of the same task and procedure with infants, allowing a direct comparison across age groups (Bulf and Valenza, 2013).

The second aim of the present study was to verify whether object-based attention is differently affected by faces even in infancy. Faces are ideal stimuli for our purpose, because they easily recruit attention since birth (Morton and Johnson, 1991; Valenza et al., 1996; Macchi Cassia et al., 2004). Recently, some infants’ studies have been devoted to investigate whether faces would modulate the deployment of visual attention differently from objects (e.g., Hunnius and Geuze, 2004; Frank et al., 2009; Gliga et al., 2009; Peltola et al., 2009). Hunnius and Geuze (2004) found that the face modulates the disengagement of attention during infancy. More recently Gliga et al. (2009) showed that at 6 months of age faces preferentially capture attention in a complex visual array containing a face among multiple visual objects. These studies have used gap/overlap and visual search paradigms to investigate how faces modulate the capture and disengagement of visual attention in the first months of life. Here, for the first time, we assessed whether faces can modulate the object-based component of visual attention in 8-month-old infants using once again the Egly et al.’s (1994) cueing task (Experiment 2).

Moreover, given that a critical factor in eliciting the deployment of object-based attention is the establishment of a viable representation (Chen, 2012), we hypothesized that a face might influence the deployment of visual attention between-objects differently in infancy compared to adulthood. Indeed, face representation change with development becoming more specialized. Infants begin to show evidence of forming face prototypes by 3 months of age (De Haan et al., 2001), and continue to undergo changes for many years before they become adult-like. For example, during development infants’ face representations become specific to familiar characteristics of faces (Turati et al., 2005) such as those belonging to own ethnic group (Pascalis et al., 2005; Kelly et al., 2007), specie (Pascalis et al., 2002), and age (Macchi Cassia et al., 2009). The use of the same cueing task presented to the adults in Experiment 1 allowed us to compare the influence of a face on object-based attention across the two ages.

## EXPERIMENT 1

Experiment 1 was addressed to verify whether faces affect adults’ object-based attention. A social species like ours depends highly on face-mediated social interactions and thus should benefit from mechanisms that allow shifting attention among faces. Therefore it is probably that, when two faces occurred in the environment, the viewer spreads his/her attentional resources between the two stimuli by broadening the focus of attention. Starting from this consideration we expected that, when upright faces will be presented, an object-based effect (i.e., faster saccadic latencies toward targets that appeared within a previously cued object than toward targets that appeared in a different object) will not occur. Conversely, we expected to obtain an object-based effect when scrambled faces will be presented. Moreover, given that previous behavioral studies with adults revealed no difference between upright and inverted faces in a face detection task (e.g., Kuehn and Jolicoeur, 1994; Brown et al., 1997; Van Rullen, 2006), we expected that object-based effect might not occur even for the inverted-faces.

### MATERIAL AND METHODS

#### Participants

Thirty-four undergraduate students participated in the experiment. All participants reported normal vision and none wore glasses or contact lenses. Four participants were excluded from the sample because of uninterpretable eye movements due to poor calibration of the point of gaze (*n* = 3), or program errors during data collection (*n* = 1). Thirty participants comprised the final sample, ranging in age from 19 to 26 years (mean age = 21 years, SD = 1.8; 24 females). Participants gave their informed consent before participating in the study. The departmental ethical committee approved the present study (code 1149–2012), and the experiment was conducted in accordance with the ethical standards of the 1964 Declaration of Helsinki and its later amendments.

#### Stimuli

**FIGURE 1:**
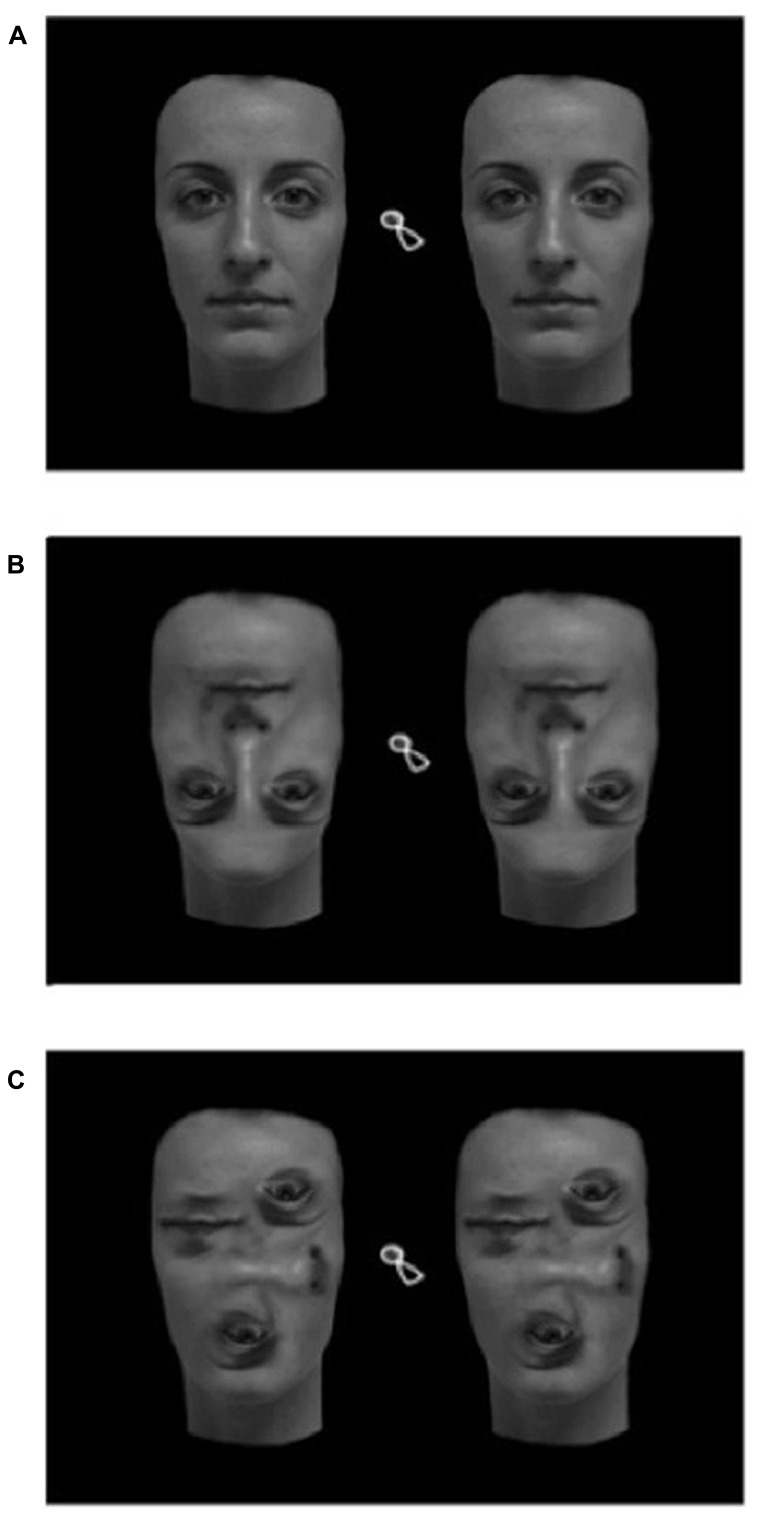
**Each stimulus was composed of two upright **(A)**, inverted **(B),** or scrambled **(C)** faces**. Original stimuli were colored.

The computer screen showed a pair of stimuli presented on a black background. The pair of stimuli may involve two upright, inverted or scrambled faces (see **Figure 1**). Colored photographs of four women’s faces were used. Each face was photographed in a frontal pose and with a neutral expression. Photographs were modified with Adobe Photoshop^®^ CS4, so that an upright face, an inverted and a scrambled version of the same face were generated. Hair was removed from each photograph. On the inverted face, only the inner portion of the face was rotated 180°. Scrambled versions were generated displaying and rearranging the inner features of the face. Stimuli measured 10 cm (9.5°) in width and 15 cm (14.3°) in height.

The cue was a red dot with a diameter of 3 cm (2.9°) and a transparency of 41%. The target was a yellow dot with the same dimension and transparency of the cue.

#### Apparatus

The stimuli were presented with E-Prime 2.0 on a 19-inch monitor with a resolution of 1024x768 pixels. A remote, pan-tilt infrared eye-tracking camera (Model 504, Applied Science Laboratories [http://www.a-s-1.com], Bedford, MA, USA) using bright-pupil technology, placed directly below the stimulus screen, recorded the participant’s eye movements at a temporal resolution of 50 Hz. Infrared light emitted from diodes on the camera was reflected back from the participant’s retina through the pupil, producing a backlit, white pupil from the corneal surface of the eye. An experimenter guided the eye-tracking camera by means of a remote control, so that the eye of the participant was always in focus. The image of the eye on a television monitor made this procedure easier. Plain curtains were hung on both sides of the testing area to prevent interference from irrelevant stimuli. Behind the curtains one computer generated the stimuli, and another one controlled the eye-tracker camera and collected the eye-movement data. To coordinate the eye-movement data with a specific stimulus display, the stimulus-generating computer sent a unique, time-stamped numerical code via a parallel port to the data-collecting computer, indicating the onset of the stimulus display and the type of stimulus display. The digital data indicating the fixation locations and change of locations of the eye (the eye movements themselves) were calculated in relation between the centroid of the pupil and the corneal reflection by using the Applied Science Laboratories’ algorithm.

Four main areas of interest (AOI) that corresponded to the possible positions of the target (top-left; bottom left; top right; bottom-right) were selected. Each AOI measured 4 cm in width and 4 cm in height and they were non-overlapping.

#### Procedure

Adults sat in a high-back chair so that their heads were resting against the back of the chair for stability. The chair was placed 60 cm distant from the stimulus monitor. Participants were told simply to look at the display, without any additional instruction.

The experimental session began with the calibration procedure that allowed the eye-tracker system to subsequently determine the precise direction of the participant’ gaze during the experimental session. The eye-tracker was calibrated by showing to participants three markers on the screen presented one by one on the top-left, on the center and on the bottom-right, and recording the eye-tracker readings for the eye-fixation location. If the recorded gaze position did not remain stable within the area covered by the calibration stimulus, a new calibration was conducted. Calibration usually lasted between 1 and 2 min. All subsequent eye data were calculated from these calibration values.

**FIGURE 2:**
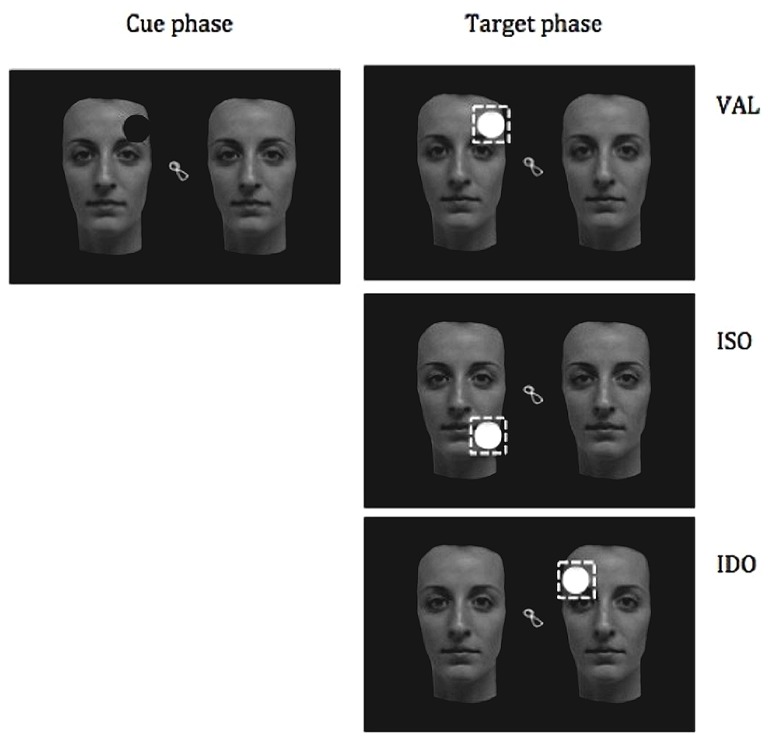
**Considering an example of the cue (originally colored of red) on the top of a left upright face, the figure shows the three possible locations of the target (originally colored of yellow) in the valid (VAL), invalid same-object (ISO), and invalid different-object (IDO) trials**. The dashed square corresponds to the area of interest for each target.

An experimental trial began with the presentation, in the middle of the screen, of a central dynamic attractor (e.g., a colored moving clown). As soon as the participants looked at this central fixation point, a pair of stimuli automatically appeared peripherally, one on the left and one on the right of the central attention-getter. After 1000 msec, a cue superimposed on the top or on the bottom of one object was presented for 100 msec. Then the central fixation was removed, and a flashing target appeared automatically after a 200 msec. Target could appear in three different locations: in the same location of the cue (valid trials; VAL), in a different location of the same object far from the cue 9 cm (ISO), or in the adjacent uncued object, placed at the same distance (9 cm) from the cue as the target in the ISO trials (IDO; see **Figure 2** for the three conditions described above). The probability of the target locations was balanced in the three conditions. The target remained visible until the participant made a saccade toward it or for a maximum of 2 s. This terminated the trial, and another trial began with the central fixation point.

A total of 72 trials (8 trials × 3 types of stimulus × 3 conditions) were administered. The left vs. right positions of the cue within each pair were counterbalanced. The presentation sequence of each trial was randomly determined and it was arranged in two blocks of presentation, so the participants could take a small break in half of the experimental session. The entire experiment lasted about 8–10 min.

### RESULTS

Software E-Prime allowed us to elaborate the raw data coming from the eye-tracker system, calculating participants’ latency to reach the AOI where the target appeared. **Table 1** shows the mean latencies and SD of the trials enclosed in the analysis (i.e., saccade that reaches the AOI where the target appeared), for each of the three displayed stimuli. A mean of 20.4 trials (SD = 9.4) for each adult was excluded by the statistical analysis for the following reasons: because adults looked outside the defined AOI corresponding to the target (*M* = 6.0, SD = 4.1) or they did not look at the central fixation point before the stimuli presentation (*M* = 6.9, SD = 4.0), or because the signal of the eye-tracker was lost during the stimuli presentation (*M* = 7.6, SD = 4.6). The final number of trials in which adults detected the target was on average 51.6 trials (SD = 9.4). Specifically, the trials included in the analysis for each condition were: *M* = 4.9 (SD = 1.3) for the VAL, *M* = 5.9 (SD = 1.8) for the ISO, *M* = 6.4 (SD = 1.5) for the IDO, when upright face was presented; *M* = 4.5 (SD = 1.6) for the VAL, *M* = 6.1 (SD = 1.7) for the ISO, *M* = 6.6 (SD = 1.5) for the IDO, when the inverted face was shown; *M* = 4.8 (SD = 1.7) for the VAL, *M* = 6.0 (SD = 1.6) for the ISO, *M* = 6.8 (SD = 1.2) for the IDO, when the scrambled face was presented.

**Table 1:** Adults’ mean latencies (SD) expressed in msec for the different target locations toward the upright, inverted, and scrambled faces

Stimuli		Target location	
Upright	324.98 (113.42)	397.86 (110.88)	391.97 (142.11)
Inverted	287.19 (139.85)	401.62 (151.03)	387.17 (146.34)
Scrambled	273.17 (130.31)	344.21 (94.23)	417.76 (170.08)

The mean response times to reach the target were initially analyzed in a two-way, within-subject analysis of variance (ANOVA) with STIMULUS (upright, inverted, scrambled), and CONDITION (VAL, ISO, IDO) as factors. The analysis revealed no significant main effect of STIMULUS *F*(2,58) = 1.65, *p* = 0.201, $\eta_{p}^{2}$ = 0.054, but a significant main effect of CONDITION *F*(2,58) = 17.12, *p* < 0.001, $\eta_{p}^{2}$ = 0.371. *Post hoc* comparisons run with the Bonferroni test revealed significant (*p* ≤ 0.001) faster responses in the VAL condition (*M* = 295.11 msec) compared to the ISO (*M* = 381.23 msec) and to the IDO condition (*M* = 398.97 msec). Thus adults’ response time revealed a conventional benefit of valid cueing for target detection. No significant effect (*p* = 0.307) between the responses in the ISO condition compared with the responses in the IDO condition was found.

**FIGURE 3:**
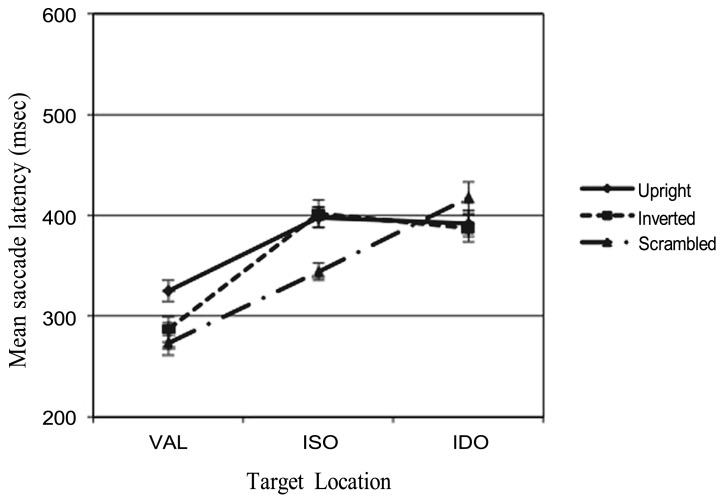
**Adults’ latencies related to the three different target locations and the three types of stimuli presented (upright, inverted, and scrambled faces)**.

Importantly, a significant interaction between factors (STIMULUS × CONDITION) *F*(4,116) = 2.54, *p* = 0.043, $\eta_{p}^{2}$ = 0.081 was found. This effect is illustrated in **Figure 3**. In particular, testing condition differences within stimuli with the Bonferroni *post hoc* test, a significant difference between ISO and IDO conditions emerged only when the scrambled face were presented (*p* = 0.005), while when the upright or the inverted face were shown no significant differences (*p* = 1.00) between ISO and IDO condition was found (see the means in **Table 1**). Besides, testing stimuli differences within condition with the Bonferroni test, two significant differences between the upright face and inverted face vs. scrambled face emerged (respectively, *p* = 0.012; *p* = 0.008) only in the ISO condition.

**FIGURE 4:**
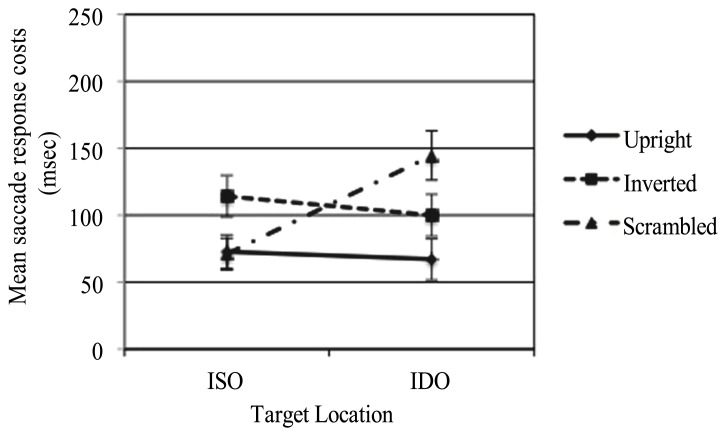
**Adults’ means saccade response costs from invalid cueing trials (given by invalid-cue latencies minus valid-cue latencies) related to the two invalid conditions (i.e., whether the stimuli that contained the target was cued or uncued) and the three types of stimuli presented (upright, inverted, and scrambled faces)**.

To better understand the effect of the cost of attention for the two kinds of invalid conditions (i.e., ISO vs. IDO) an analysis of saccadic responses costs was conducted. Costs were calculated by subtracting from the mean of each invalid condition the mean for the corresponding valid condition. A two-way, within-subject ANOVA with STIMULUS (upright, inverted, scrambled), and CONDITION (ISO, IDO) as factors was run. The analysis revealed no significant main effect of STIMULUS *F*(2,58) = 0.95 *p* = 0.394, $\eta_{p}^{2}$ = 0.032, and CONDITION *F*(1,29) = 2.85, *p* = 0.102, $\eta_{p}^{2}$ = 0.089. A significant interaction between factors (STIMULUS × CONDITION) *F*(2,58) = 4.62, *p* = 0.014, $\eta_{p}^{2}$ = 0.138 was found. This effect is illustrated in **Figure 4**. In particular, the Bonferroni *post hoc* test highlighted a significant difference between ISO and IDO conditions only when the scrambled face was presented (*p* = 0.002), while when the upright or the inverted faces were shown no significant differences was found (respectively, *p* = 0.798; *p* = 470).

### DISCUSSION

Adults showed a space-based effect, with slower latencies to detect targets in the invalid conditions than in the valid conditions irrespective to all the stimuli presented (upright, inverted, or scrambled faces). Interestingly, faster latencies (i.e., smaller cost) in the ISO than in the IDO were found only when scrambled faces were presented. This result provides evidence for an object-based effect when scrambled faces were presented, such that when a given location is cued, the effects of that cue spread to other locations within the cued object, leading to a smaller cost to shift attention to different locations of the same object than to different-object locations when the spatial distance is preserved. The object-based effect was not obtained in the upright and inverted faces. Probably, the great experience that adults have with faces exposed in different poses or upside-down allows them to easily detect the inverted face as a face. Overall, our findings provide evidence that the upright/inverted faces and scrambled faces differently affect the object-based attention in adulthood.

## EXPERIMENT 2

Adults have the remarkable ability to detect faces, even if faces are composed of objects such as an arrangement of fruits, vegetables or rocks (Bruce and Young, 1986), or they are two-tone Mooney face (Kanwisher et al., 1998). This extraordinary ability is acquired during development and is dependent upon visual experience.

The Experiment 2 was designed to investigate whether faces modulate object-based attention even if the exposure to faces has been not abundant. To this aim, upright, and inverted faces were presented to 8-month-old infants. Differently from Experiment 1, we did not present a scrambled face condition to infants because, since it is unlikely that scrambled faces are present in an ecological environment, we reasoned that infants’ and adults’ performance would not have been different. Instead, the experience accumulated with inverted faces might be different from infancy to adulthood. In relation to adults, the infants’ experience with faces is much more frequently reserved to the upright faces, because when adults are providing stimulation to infants, their faces are seen by the infants in their canonical orientation and therefore infants’ early representation of faces contains information about faces that is orientation-specific. This evidence is supported by some infants’ studies in which an advantage of upright faces over inverted faces in triggering infants’ attention has been demonstrated using gap/overlap paradigms (Otsuka et al., 2012).

Given that infants’ experience with faces is much more frequently reserved to the upright faces, the object-based attention might be affected in a different manner in 8-month-old infants when upright or inverted faces are presented. As adults, we expected an absence of the object-based effect for upright faces. Differently from the adults’ findings, in which the object-based attention behaves similarly for the upright and inverted faces, we expected to found an object-based effect with the inverted faces.

### MATERIAL AND METHODS

#### Participants

Forty-two healthy and full-term infants participated in the experiment. Thirty infants (13 males and 17 females) with a mean age of 8 months and 9 days (mean age = 249 days, SD = 5.35, range = 240 days to 260 days) comprised the final sample. Twelve infants were observed but not included in the analyses, due to fussiness or drowsiness (4), program errors during data collection (1), excessive movement of the infant, such that we were unable to record eye movements (3), or poor calibration in detecting with the eye-tracker the infant’s gaze direction in a reliable way (4). Infants were recruited from a database of new parents, and parents were contacted by letter and telephone. Infants were tested only after their parents had given their informed consent. The departmental ethical committee approved the present study (code 1149–2012), and the experiment was conducted in accordance with the ethical standards of the 1964 Declaration of Helsinki and its later amendments.

#### Stimuli and apparatus

The stimuli and the apparatus were identical to those used in Experiment 1, with the exception that only upright and inverted faces were presented. Scrambled versions of the face were removed in order to reduce the total number of trials and therefore the time course of the experiment.

#### Procedure

The procedure was the same as with adults, but infants sat in an infant car seat placed 60 cm distant from the stimulus monitor. Parents usually were seated behind the infant seat, slightly moved randomly to the right or left side of the infant, so they could see the monitor and be close to their baby. The room lights were first lowered, and the infants shown a dynamic cartoon with a musical soundtrack to engage his or her interest toward the predetermined locations, as the experimenter directed the pupil camera toward the participants’ eye with the remote control.

Removing a pair of stimuli (i.e., the scrambled faces), the number of trials was lower than in Experiment 1. A total of 48 trials (8 trials × 2 types of stimuli × 3 conditions) were administered for infants. The left vs. right positions of the cue within each pair were counterbalanced. The presentation sequence of each trial was randomly determined and it was arranged in two blocks of presentation, so the infants could take a break in half of the experimental session. The entire experiment lasted about 15–20 min, depending on the state of the infant.

### RESULTS

Software E-Prime allowed us to elaborate the raw data coming from the eye-tracker system, calculating participants’ latency to reach the AOI where the target appeared. **Table 2** shows the mean latencies and SD for all infants’ trials enclosed in the analysis (i.e., saccade that reaches the AOI), for each of the two displayed pair of stimuli.

**Table 2:** Infants’ mean latencies (SD) expressed in msec for the different target locations toward the upright and inverted faces.

Stimuli	Target location		
	VAL	ISO	IDO
Upright	420.00 (151.75)	553.36 (153.85)	556.98 (193.66)
Inverted	366.87 (131.47)	468.99 (122.74)	580.39 (151.04)

A mean of 20.2 trials (SD = 7.1) for each infant was excluded by the statistical analysis for the following reasons: because infants looked outside the defined AOI corresponding to the target (*M* = 4.4; SD = 2.4), or they did not look at the central fixation point before the stimuli presentation (*M* = 6.7; SD = 3.3), or because the signal of the eye-tracker was lost during the stimuli presentation (*M* = 9.0; SD = 4.0). The final number of trials in which infants detected the target was on average 27.8 trials (SD = 7.1). Specifically, the trials included in the analysis for each condition were: *M* = 4.8 (SD = 1.7) for the VAL, *M* = 4.7 (SD = 1.5) for the ISO, *M* = 4.7 (SD = 1.5) for the IDO, when upright face was presented; *M* = 4.2 (SD = 1.6) for the VAL, *M* = 4.7 (SD = 1.4) for the ISO, *M* = 4.3 (SD = 1.7) for the IDO, when the inverted face was shown.

**FIGURE 5:**
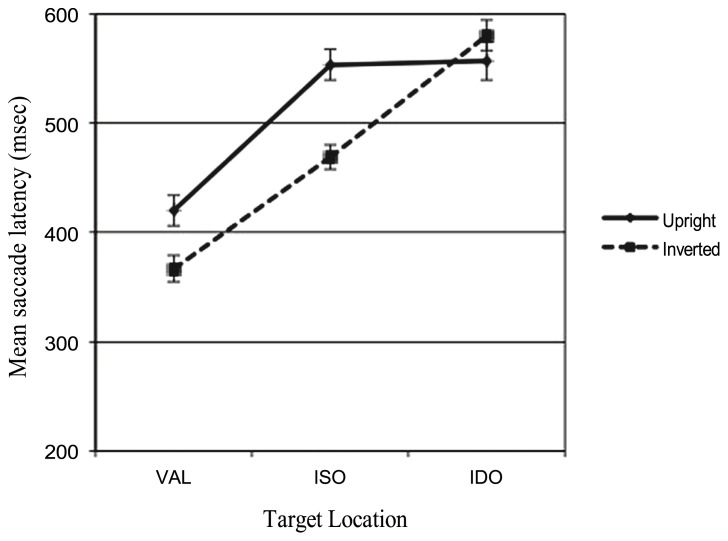
**Infants’ latencies related to the three different target locations and the two types of stimuli presented (upright and inverted faces)**.

The mean response times to reach the target were initially analyzed in a two-way, within-subject ANOVA with STIMULUS (upright, inverted), and CONDITION (VAL, ISO, IDO) as factors. The analysis revealed no significant main effect of STIMULUS *F*(1,29) = 2.05, *p* = 0.163, $\eta_{p}^{2}$ = 0.066, but a significant main effect of CONDITION *F*(2,58) = 21.96, *p* < 0.001, $\eta_{p}^{2}$ = 0.431. *Post hoc* comparisons run with the Bonferroni test revealed significant faster responses (*p* < 0.001) in the VAL condition (*M* = 393.44 msec) compared to the ISO (*M* = 511.18 msec) and to the IDO condition (*M* = 568.68 msec). This comparison showed that the conventional benefit of valid cueing was observed for target detection. No significant effect between the infants’ saccadic responses recorded in the ISO condition compared with those in the IDO condition was found (*p* = 0.079). Analysis revealed also a significant interaction between the factors (STIMULUS × CONDITION)* F*(2,58) = 3.23, *p* = 0.047, $\eta_{p}^{2}$ = 0.100. This effect is illustrated in **Figure 5**. In particular, testing condition differences within stimuli with the Bonferroni *post hoc* test, a significant difference between ISO and IDO conditions emerged only when the inverted face were presented (*p* = 0.001), while when the upright face were shown no significant differences (*p* = 1.00) between ISO and IDO condition was found (see the means in **Table 2**). Besides, testing stimuli differences within condition with the Bonferroni test, a significant difference between the upright face vs. inverted face emerged (*p* = 0.021) only in the ISO condition.

**FIGURE 6:**
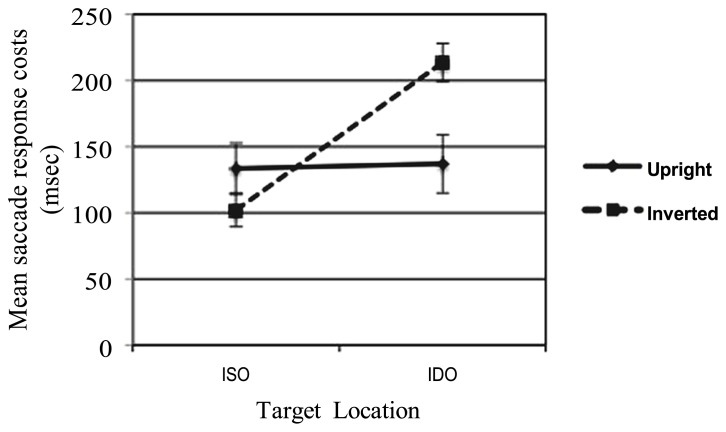
**Infants’ means saccade response costs from invalid cueing trials (given by invalid-cue latencies minus valid-cue latencies) related to the two invalid conditions (i.e., whether the stimuli that contained the target was cued or uncued) and the two types of stimuli presented (upright and inverted faces)**.

As in the previous Experiment, the effect of the cost of attention for the invalid conditions (relative to the corresponding valid trial baseline) was deeply analyzed. A two-way ANOVA with STIMULUS (upright, inverted), and CONDITION (ISO, IDO) as factors was conducted on the resulting data. The analysis revealed no significant main effect of STIMULUS *F*(1,29) = 0.41, *p* = 0.527, $\eta_{p}^{2}$ = 0.014, but a significant main effect of CONDITION *F*(1,29) = 5.47, *p* = 0.026, $\eta_{p}^{2}$ = 0.159. The cost for detecting the target in the IDO condition (*M* = 175.25 msec) was greater than that for the ISO condition (*M* = 117.47 msec). This critical comparison reflects a time cost for shifting attention between-objects, thus demonstrating an object-based effect. Intriguingly, analysis revealed also a significant interaction between the factors (STIMULUS × CONDITION) *F*(1,29) = 5.43, *p* = 0.027, $\eta_{p}^{2}$ = 0.158. In particular, the Bonferroni *post hoc* test highlighted a significant difference between the cost for the ISO and IDO conditions only when the inverted face were presented (*p* < 0.001), while when the upright face were shown no significant cost differences (*p* = 0.927) was found. This effect, illustrated in **Figure 6**, provide evidence that in infancy upright and inverted faces differently affect the cost to shift attention between-objects.

### DISCUSSION

Results showed slower latencies to detect targets in the invalid conditions than to the valid conditions (space-based effect), irrespective to the type of stimulus. Intriguingly, faster latencies (i.e., smaller cost) in the ISO than in the IDO were found only when inverted faces were presented. This object-based effect was not confirmed in the upright faces, suggesting that the object component of attention is affected by the type of stimulus even in infancy.

## A COMPARISON BETWEEN ADULTS’ AND INFANTS’ PERFORMANCE

Adults have undeniably more experience with face stimuli than infants, and therefore it is reasonable to expect that stimuli used in the current study may constrain in a different way adults’ and infants’ object-based attention. In order to better understand the effect of the stimulus on object-based attention between the two ages we investigated whether adults and infants paid a different cost in the two invalid conditions (i.e., ISO e IDO) respectively for upright and inverted face. The cost of attention (i.e., the difference between the mean of each invalid condition and the mean for the corresponding valid condition) was analyzed. The analysis revealed a significant interaction between CONDITION and AGE factors *F*(1,58) = 5.87, *p* = 0.019, $\eta_{p}^{2}$ = 0.092. The Bonferroni *post hoc* test highlighted a significant difference (*p* = 0.020) between infants and adults in the IDO (*M* = 175.25 msec vs. *M* = 83.49 msec) condition. This result suggested that infants are slower to the detect the target in the IDO condition than adults.

**FIGURE 7:**
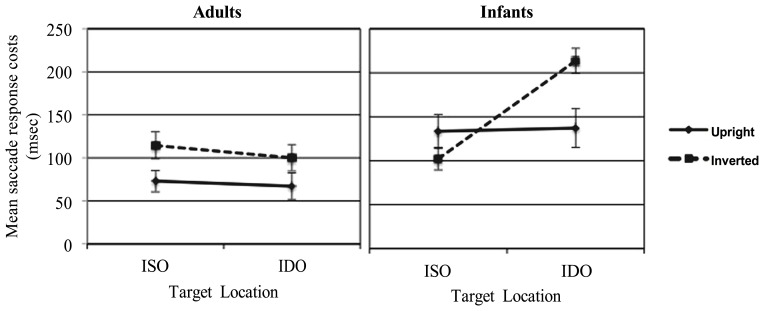
**Adults’ and infants’ saccade response costs related to the ISO and IDO conditions for the upright and inverted face stimuli**.

The analysis revealed also a significant interaction between STIMULUS × CONDITION × AGE *F*(1,58) = 4.16, *p* = 0.046, $\eta_{p}^{2}$ = 0.067. The Bonferroni *post hoc* test highlighted, only in the infants group, a significant difference (*p* < 0.001) between ISO and IDO conditions (respectively, *M* = 102.12 msec vs. *M* = 213.52 msec) for the inverted stimulus. The Bonferroni *post hoc* test highlighted also a significant difference (*p* = 0.010) between infants and adults (respectively, *M* = 99.99 msec vs. *M* = 213.52 msec) in the IDO condition only for the inverted stimulus. Altogether these findings illustrated in **Figure 7**, suggest that the cost of attention varied between the two groups of age according to the type of stimulus and to the condition. Indeed, when upright faces are presented adults and infants did not show an object-based effect. Conversely only for 8-month-old infants, but not for adults, the inverted face determines an object-based effect.

## GENERAL DISCUSSION

Object-based attention operates on perceptual objects, opening the possibility that the “costs and benefits” humans have to pay to move attention between-objects might be affected by the type of stimulus. In the present study, we investigated the role of face or non-face stimuli in affecting adults’ and infants’ object-based attention.

To our knowledge, this is the first demonstration that the cost to spread attention between stimuli is different if objects are faces or non-faces stimuli. Data revealed slower latencies to detect targets in the invalid conditions (i.e., when the target did not appear in the cued location) than in the valid conditions (i.e., when the target appeared in the cued location) both for adults (Experiment 1) and infants (Experiment 2). This result suggests that attentional selection favors location-based attention because the responses were faster at the cued location, supporting the well-known benefit of valid cueing for target detection (space-based effect). This result suggests also that, at least in the present study, the space-based effect of attention is not affected by the salience of the stimulus given that target detection was always faster in valid condition than in invalid condition both for face and non-face stimuli (but see Theeuwes and Van der Stigchel, 2006; Langton et al., 2008 for the influence of faces on spatial attention when different attentional tasks were used).

Our findings reveal also that when the target appeared on scrambled faces (Experiment 1 with adults), or on inverted faces (Experiment 2 with infants), participants paid a smaller cost (faster latencies) in the ISO condition than in the IDO condition, providing evidence for an object-based effect such that when a given location is cued, the effect of that cue is spread to other locations within the cued object, so the entire object benefits perceptually, speeding performance even for the uncued regions of that object. Note that the distance between the cue and the target that appeared in the cued object was equal to the distance between the cue and the target that appeared in the uncued object. The control of the distance between cue and target in the ISO and IDO conditions is fundamental in this case, considering that the allocation of attention should be weaker the farther away the target appeared from the cued location (Downing and Pinker, 1985). Crucially, the object-based effect is not obtained when the target appeared on the upright faces, meaning that both adults (Experiments 1) and infants (Experiments 2) pay the same attentional cost to shift attention within the cued face or between the cue and the uncued face.

Overall, our findings open two questions: (1) Why does the object–based effect disappear when attention has to be shifted between upright faces? and (2) Why does the inverted face determine an object-based effect only in 8-month-old infants, but not in adults? The answers to these questions are critical for the interpretation of the findings of this study. In the following sections we will try to answer to these questions separately.

## WHY DOES THE OBJECT-BASED EFFECT DISAPPEAR WHEN ATTENTION HAS TO BE SHIFTED BETWEEN FACES?

Faces have a special capacity to recruit attention. From birth infants preferentially orient to and spend more time looking at a face rather than a non-face stimulus (Morton and Johnson, 1991; Valenza et al., 1996; Macchi Cassia et al., 2004). The preference for schematic face stimuli declines after the first month of life, but older infants display preference for faces when tested with more realistic faces (Morton and Johnson, 1991). Moreover very early infants learn that faces are communicative stimuli and that it is useful to pay attention to them. Consequently, it is plausible that, when more than one face is present in the environment, the viewer’s focus of attention is enlarged in order to contain all the faces with which they might interact. We explain our results assuming that the size of the adults’ and infants’ attentional focus have been differently modified when face or non-face stimuli have been presented.

This explanation is in line with those studies that have investigated how the size of attentional focus affects the detection of a target. Several studies have demonstrated that the RTs to the target are slower when the target appeared outside the attentional focus in comparison with its appearance inside the attentional focus (Facoetti and Molteni, 2001; Ronconi et al., 2013). This effect is called *attentional gradient effect* (Ronconi et al., 2013) and it might occur when two inverted (in case of infants) or scrambled (in case of adults) faces were shown. Indeed, when non-face stimuli (inverted or scrambled faces) are presented the size of the attentional focus might be narrowed to the cued stimulus and therefore we obtained a slower response time in the IDO compared to the ISO condition because the target appeared inside the viewer’s focus in the cued stimulus (i.e., ISO condition), and outside the viewer’s focus in the uncued stimulus (i.e., IDO condition). In contrast when the size of the attentional focus is enlarged, the *attentional gradient effect* is reduced or nullified because the target is presented inside the focus regardless target position. This is exactly what might occur when upright faces are presented. We did not obtain an object-based effect when the two upright faces were presented, because the viewer enlarged the size of his/her focus of attention in order to attend to both the stimuli, so the target appeared always inside the viewer’s attentional focus, both if it appeared in the cued (i.e., ISO condition) or in the uncued face (i.e., IDO condition). In other words, when upright faces are presented adults and infants spread their attention between the two stimuli by broadening the focus of attention. In this way, the target appears always inside their attentional focus and the difference between the ISO and the IDO condition disappears. Future researches might be addressed to investigate more directly the relationship between object-based attention and attentional focusing mechanisms.

The interpretation that the attentional focus size is enlarged for faces is also supported by the results indicating slower response times to reach the target in the ISO condition when faces were presented compared to the response times to reach the target when scrambled (in case of adults) or inverted (in case of infants) faces were presented. This result seems in line with the so called *cue size effect,* demonstrated by several studies that examined the extention of the attentional focus (e.g., Castiello and Umiltà, 1990; Benso et al., 1998; Mizuno et al., 1998; Turatto et al., 2000). This effect posits an inverse relation between the extent of the attentional focus and the efficiency of processing within its borders. That is, in processing a visual stimulus, the concentration of attentional resources inside a small area leads to faster reaction time than does the concentration inside a large area, since a larger attentional focus size requires the exploration of a wider area for target detection.

## WHY DOES THE INVERTED FACE DETERMINE AN OBJECT-BASED EFFECT ONLY IN 8-MONTH-OLD INFANTS, BUT NOT IN ADULTS?

A further interesting result of this study concerns the difference between adults’ and infants’ object-based effect in the inverted face condition. Findings reveal that the inverted face determines an object-based effect in 8-month-old infants, but not in adults, suggesting that infants treat inverted faces as non-face objects while adults treat inverted faces as faces. This finding seems in contrast with an extensive literature in adults’ face processing revealing that the process involved in face recognition is qualitatively different from those involved in the recognition of other kinds of objects. However, when adults are asked to simply detect a face in a complex visual array, no differences was find between upright and inverted faces (Kuehn and Jolicoeur, 1994; Brown et al., 1997; Van Rullen, 2006). Functional brain imaging investigations of the human brain have complemented the evidence from behavioral studies, showing that inversion of a face disrupts the ability to recognize the face but not the ability to detect a face, that is, to see that a face is present (Kanwisher et al., 1997). Thus it seems that in adulthood inversion is detrimental only when additional processing is required (e.g., identification). Given that in our study it was not requested to recognize a face, it is not surprising that adults manifested similar attentional performance when upright or inverted faces are presented. Probably, the great experience that adults have with the face exposed in different poses, allows them to easily identify the inverted face as a face.

Even if infants see faces in different poses, it is likely that when adults are providing social stimulation to infants their faces are seen by the infants in their canonical orientations. So the difference between adults’ and infants’ performance with inverted faces might be due to their different experience with these stimuli. The assumption that subject’s experience can constrain the deployment of object-based attention is supported by a study carried out by Li and Logan (2008) in which skilled Chinese readers showed object effect when they switched attention between Chinese characters that were part of a word, relative to parts of two words. These results showed that objects defined by subjects’ knowledge – in this case, lexical information – can constrain the deployment of attention (Li and Logan, 2008).

In conclusion, for the first time the current experimental study demonstrated that the object-based attention is modulated by the type of the stimulus and by the experience acquired by the viewer with different objects.

## AUTHOR CONTRIBUTIONS

Eloisa Valenza and Hermann Bulf designed research; Hermann Bulf and Laura Franchin performed research; Laura Franchin codified and analyzed data; Eloisa Valenza and Hermann Bulf wrote the Introduction and General Discussion; Laura Franchin wrote the Methods and Results.

## Conflict of Interest Statement

The authors declare that the research was conducted in the absence of any commercial or financial relationships that could be construed as a potential conflict of interest.
